# Identification of cholesterol metabolism-related subtypes in nonfunctioning pituitary neuroendocrine tumors and analysis of immune infiltration

**DOI:** 10.1186/s12944-023-01883-3

**Published:** 2023-08-10

**Authors:** Tianshun Feng, Pengwei Hou, Shuwen Mu, Yi Fang, Xinxiong Li, Ziqi Li, Di Wang, Li Chen, Lingling Lu, Kunzhe Lin, Shousen Wang

**Affiliations:** 1 Department of Neurosurgery, Dongfang Affiliated Hospital of Xiamen University, School of Medicine, Xiamen University, Fuzhou, China; 2 Department of Neurosurgery, Fuzhou 900th Hospital, Fuzong Clinical Medical College of Fujian Medical University, Fuzhou, China; 3 Department of Neurosurgery, Peking Union Medical College Hospital, Chinese Academy of Medical Sciences and Peking Union Medical College, Beijing, China; 4 Department of General Surgery, School of Medicine, Dongfang Affiliated Hospital of Xiamen University, Xiamen University, Fuzhou, China; 5 Department of Molecular Pathology, Fujian Cancer Hospital, Clinical Oncology School of Fujian Medical University, Fuzhou, China; 6 Fuzong Clinical Medical College of Fujian Medical University, Fuzhou, China

**Keywords:** Cholesterol metabolism, Immune infiltration, Nonfunctioning pituitary neuroendocrine tumor, Cavernous sinus invasion

## Abstract

**Objective:**

This study aimed to investigate the role of cholesterol metabolism-related genes in nonfunctioning pituitary neuroendocrine tumors (NF-PitNETs) invading the cavernous sinus and analyze the differences in immune cell infiltration between invasive and noninvasive NF-PitNETs.

**Methods:**

First, a retrospective analysis of single-center clinical data was performed. Second, the immune cell infiltration between invasive and noninvasive NF-PitNETs in the GSE169498 dataset was further analyzed, and statistically different cholesterol metabolism-related gene expression matrices were obtained from the dataset. The hub cholesterol metabolism-related genes in NF-PitNETs were screened by constructing machine learning models. In accordance with the hub gene, 73 cases of NF-PitNETs were clustered into two subtypes, and the functional differences and immune cell infiltration between the two subtypes were further analyzed.

**Results:**

The clinical data of 146 NF-PitNETs were evaluated, and the results showed that the cholesterol (*P* = 0.034) between invasive and noninvasive NF-PitNETs significantly differed. After binary logistic analysis, cholesterol was found to be an independent risk factor for cavernous sinus invasion (CSI) in NF-PitNETs. Bioinformatics analysis found three immune cells between invasive and noninvasive NF-PitNETs were statistically significant in the GSE169498 dataset, and 34 cholesterol metabolism-related genes with differences between the two groups were obtained 12 hub genes were selected by crossing the two machine learning algorithm results. Subsequently, cholesterol metabolism-related subgroups, A and B, were obtained by unsupervised hierarchical clustering analysis. The results showed that 12 immune cells infiltrated differentially between the two subgroups. The chi-square test revealed that the two subgroups had statistically significance in the invasive and noninvasive samples (*P* = 0.001). KEGG enrichment analysis showed that the differentially expressed genes were mainly enriched in the neural ligand–receptor pathway. GSVA analysis showed that the mTORC signaling pathway was upregulated and played an important role in the two-cluster comparison.

**Conclusion:**

By clinical data and bioinformatics analysis, cholesterol metabolism-related genes may promote the infiltration abundance of immune cells in NF-PitNETs and the invasion of cavernous sinuses by NF-PitNETs through the mTOR signaling pathway. This study provides a new perspective to explore the pathogenesis of cavernous sinus invasion by NF-PitNETs and determine potential therapeutic targets for this disease.

**Supplementary Information:**

The online version contains supplementary material available at 10.1186/s12944-023-01883-3.

## Introduction

NF-PitNETs account for approximately 14–54% of PitNETs [1]. However, the clinical term “nonfunctional” is not a diagnosis but rather a description of a clinical scenario with many differential diagnoses [2]. Such tumors have no clinical evidence of abnormally high pituitary hormones. Gonadotropin tumors are the most common type of NF-PitNETs, accounting for approximately 70–75% [3, 4]. Meanwhile, NF-PitNETs also include silent corticotroph tumors, silent PIT-1 lineage tumors, and Null cell tumors [2, 5, 6]. NF-PitNETs are usually bulky, and tumor invasion of the cavernous sinus has been shown to be the main cause of incomplete tumor resection and postoperative recurrence [7]. Therefore, understanding the pathogenesis of CSI by NF-PitNETs could help discover new therapeutic ideas.

Cholesterol, one of the components of the cell membrane, maintains the integrity and fluidity of the cell membrane [8] and is involved in the proliferation and migration of cells. Currently, increasing research supports the importance of reprogramming cholesterol metabolism in the progression of cancer. For example, cholesterol could trigger and promote many cancers, such as gastric cancer, prostate cancer, and breast cancer, by forming bile acids or covalently modifying proteins and regulating signaling pathways involved in tumorigenesis and cancer progression [9, 10].

In addition to containing cancer cells, tumors contain various immune effector cells and immunosuppressive cells, collectively known as tumor-infiltrating immune cells, and their function is affected by cancer type and stage [11]. However, cholesterol and its metabolites also affect the infiltration of immune cells in the tumor microenvironment (TME) [12].

Previous studies are limited to the exploration of cholesterol metabolism in the pathogenesis of PitNETs. For example, cholesterol metabolism has been reported to play a potential tumorigenic role in PitNETs progression by activating hedgehog signaling [13]. The study creatively explores the association between cholesterol metabolism-related genes and CSI in PitNETs. The present study aimed to understand the pathogenesis of CSI by NF-PitNETs from the perspective of cholesterol metabolism, and discover hub cholesterol metabolism-related genes, which may be significant markers for the diagnosis of invasiveness and provide potential therapeutic modalities for NF-PitNETs.

## Methods

### Patient cohort

In this study, clinical data of patients with NF-PitNETs from January 2016 to January 2022 in the Department of Neurosurgery, Dongfang Affiliated Hospital of Xiamen University, were retrospectively collected. The inclusion criteria were as follows: 1). first NFPA surgical resection; 2). complete surgical records, preoperative imaging data, and blood routine biochemical data. The exclusion criteria were as follows: (1) genetic history of familial dyslipidemia; (2) smoking and drinking history; (3) taking drugs that change the cholesterol content in the blood.

Finally, the clinical data of 146 patients were collected in this study, including age, gender, height, and weight, magnetic resonance findings by 3.0T magnetic resonance scanner, preoperative blood biochemical parameters, and surgical records. In this study, the Knosp grades classification was assessed by three neurosurgeons. Ultimately, the study defined Konsp grades 0–2 as a noninvasive NF-PitNETs and Knosp grades 3–4 as an invasive NF-PitNETs [14]. The hospital’s Ethics Committee approved the study, and a written informed consent for all clinical procedures and studies was given by the patients or their families.

### Acquisition of datasets and cholesterol-related genes

This study was performed in the GEO database (http://www.ncbi.nlm.nih.gov/geo/), and the GSE169498 dataset and GSE51618 were obtained [15]. The GSE169498 dataset contained 49 nonfunctioning CSI tumor samples and 24 nonfunctioning noninvasive tumor samples, which were used for the investigation of NF-PitNETs invasive diagnostic characteristics, immune cell infiltration analysis, and cholesterol-related subtype identification. In this study, seven nonfunctioning pituitary adenoma specimens from the GSE51618 dataset, including four noninvasive and three invasive samples, were used to validate hub genes. The datasets were normalized using the limma R package for expression matrices.

In the Molecular Signatures Database (http://www.gsea-msigdb.org/gsea/msigdb/), 155 cholesterol metabolism-related genes were obtained. And after intersecting with the gene set of GSE169498, the expression matrix of 132 cholesterol metabolism-related genes was finally obtained.

### Immune cell infiltration analysis

By using the ssGSEA algorithm in the GSVA package, the relative infiltration abundance of 28 immune cells between invasive and non-invasive NF-PitNETs were estimated, and immune cells with differential infiltration between the two groups were obtained.

### Differential expression of cholesterol-related genes and protein–protein interaction (PPI) analysis

Analysis of 132 genes by using the limma R package yielded 34 cholesterol metabolism-related genes that were statistically different in the GSE169498 expression matrix (*P* < 0.05). Subsequently, the PPI networks of 34 cholesterol metabolism genes were obtained in the STRING database, and the genes were analyzed using the MCODE plug-in in Cytoscape after removing those genes without interaction networks.

### Machine learning

In this study, 34 cholesterol metabolism genes were further screened using two machine learning algorithms. Random forest (RF) is a machine learning model containing multiple decision trees with lower generalization error and classification effect [16]. Support vector model (SVM) is a classification method used to obtain the optimal hyperplane between samples [17]. First, the “pROC” R package was used to analyze the receiver operating characteristic (ROC) of the two models and evaluate the reliability of the models. Subsequently, the screening results of the RF model and SVM were intersected to obtain hub genes. The “circlize” R package was used to visualize the links between the hub genes and analyze the relationship between these genes and 28 immune cells. Finally, the ability of intersection genes to identify aggressive pituitary neuroendocrine tumors in the test set was evaluated.

### Subgroup analysis of genes involved in cholesterol metabolism

On the basis of the expression matrix of hub genes, an unsupervised hierarchical clustering analysis of the GSE169498 dataset was performed using the “ConsensusClusterPlus” R package, followed by principal components analysis (PCA) visualization. Chi-square tests were performed to analyze the relationship of subgroup results with invasiveness and noninvasiveness. For the exploration of the differences in immune cell infiltration between different cholesterol-associated subgroups and differences in function between the two groups, ssGSEA analysis and GSVA analysis of two cholesterol-associated subgroups were performed. Finally, the relationship between 12 genes and 28 immune cells across subgroups was assessed. The differentially expressed genes (DEGs) between the two cholesterol-associated subpopulations were identified with criteria | log2fold change (FC) | > 2 and adjusted *P-*values < 0.05 and by using volcano plots. Through the R package “clusterProfiler,” Gene Ontology (GO) analysis and Kyoto Encyclopedia of Genes and Genomes (KEGG) enrichment analysis were performed to explore the function of DEGs between the two subsets.

### Statistical analysis

In this study, SPSS 26 and R 4.2.2. were used to complete the statistical and data analyses. Continuous variables conforming to normal distribution were expressed as mean ± standard deviation and compared using t-test, and count variables not conforming to normal distribution were expressed as median and compared using Mann–Whitney U test. Statistical differences between the two groups were assessed using the Wilcox test. Spearman’s correlation analysis was applied to analyze the relationship between the expression levels of genes related to cholesterol metabolism and immune cells. Chi-square test was performed to analyze statistical differences between the cholesterol-associated subgroups and different groups of samples from the test set. *P*-values less than 0.05 were considered statistically significant.

## Results

### Clinical features

A total of 146 patients with NF-PitNETs were collected (Table 1). Among them, 87 (59.6%) patients were male and 59 (40.4%) were female. According to the results of preoperative hormone tests and postoperative immunohistochemistry, 106 patients (72.6%) had gonadotropin tumors, 16 patients (11.0%) had silent corticotroph tumors, 6 patients (4.1%) had Null cell tumors, and 18 patients (12.3%) had other silent PIT-1 lineage tumors. In terms of Knosp grades, 18 patients (12.3%) had grade 0–1, 59 patients (40.4%) had grade 2, 36 patients (24.7%) had grade 3, and 33 patients (22.6%) had grade 4.

**Table 1:** Basic clinical data statistics

Characteristic	No./Total(%)
Number of patients	146
Age	55.13 ± 11.93
Gender	
Male	87(59.6)
Female	59(40.4)
Type	
gonadotrope tumors	106(72.6)
silent corticotroph tumors	16 (11.0)
null cell tumors	6 (4.1)
other silent PIT-1 lineage tumors	18 (12.3)
Knosp Grade	
0–1	18(12.3)
2	59(40.4)
3	36(24.7)
4	33(22.6)

As shown in Table 2, the results suggest no significant differences in age, gender, BMI grade, high-density lipoprotein, apolipoprotein A1, apolipoprotein B, and other factors in blood biochemical parameters between the two groups. Patients with invasive NF-PitNETs of the cavernous sinus had significantly higher Knosp grade (*P* < 0.001), cholesterol concentration in biochemical parameters (*P* = 0.034) than those with noninvasive NF-PitNETs. Binary logistic regression showed that cholesterol was an independent risk factor for CSI (Table 3).

**Table 2:** Characteristics in Cavernous Sinus Invasive and Noninvasive NF-PitNETs

Factor	CSI	No CSI	*P* value
Age	55.30 ± 11.05	54.97 ± 12.74	0.868
Gender			0.765
Male	42(60.9%)	45(58.4%)	
Female	27(39.1%)	32(41.6%)	
BMI	23.92 ± 3.68	24.25 ± 3.42	0.584
Knosp Grade			< 0.001
0–1	0	18(12.3%)	
2	0	59(40.4%)	
3	36(24.7%)	0	
4	33(22.6%)	0	
Cholesterol (mmol/L)	5.12 ± 0.89	4.78 ± 1.00	0.034
Low-density lipoprotein(mmol/L)	3.34 ± 0.91	3.02 ± 0.78	0.025
High density lipoprotein(mmol/L)	1.10 ± 0.31	1.14 ± 0.40	0.516
Apolipoprotein A1(g/L)	1.14 ± 0.25	1.16 ± 0.28	0.592
Apolipoprotein B(g/L)	1.11 ± 0.24	1.02 ± 0.27	0.038
Apolipoprotein A1/B	1.06 ± 0.31	1.22 ± 0.45	0.017
cholesterol metabolism-related subtypes			0.001
A	23(92.00%)	2(8.00%)	
B	26(54.17%)	22(45.83%)	

NF-PitNETs : Nonfunctioning Pituitary neuroendocrine tumors

CSI:Cavernous Sinus Invasive

**Table 3:** Multivariate logistic regression analysis of factors associated with CSI in nonfunctioning pituitary neuroendocrine tumors

Clinical Factors	Regression coefficient	*P* value	OR	95% confidence interval	
				Lower Limit	Upper Limit
Weight	-0.009	0.637	0.991	0.957	1.027
Height	1.516	0.52	4.553	0.045	463.221
Cholesterol	0.384	0.035	1.468	1.027	2.098

Note: OR: Odds ratio

### Thirty-four cholesterol metabolism-related genes

**Fig. 1:**
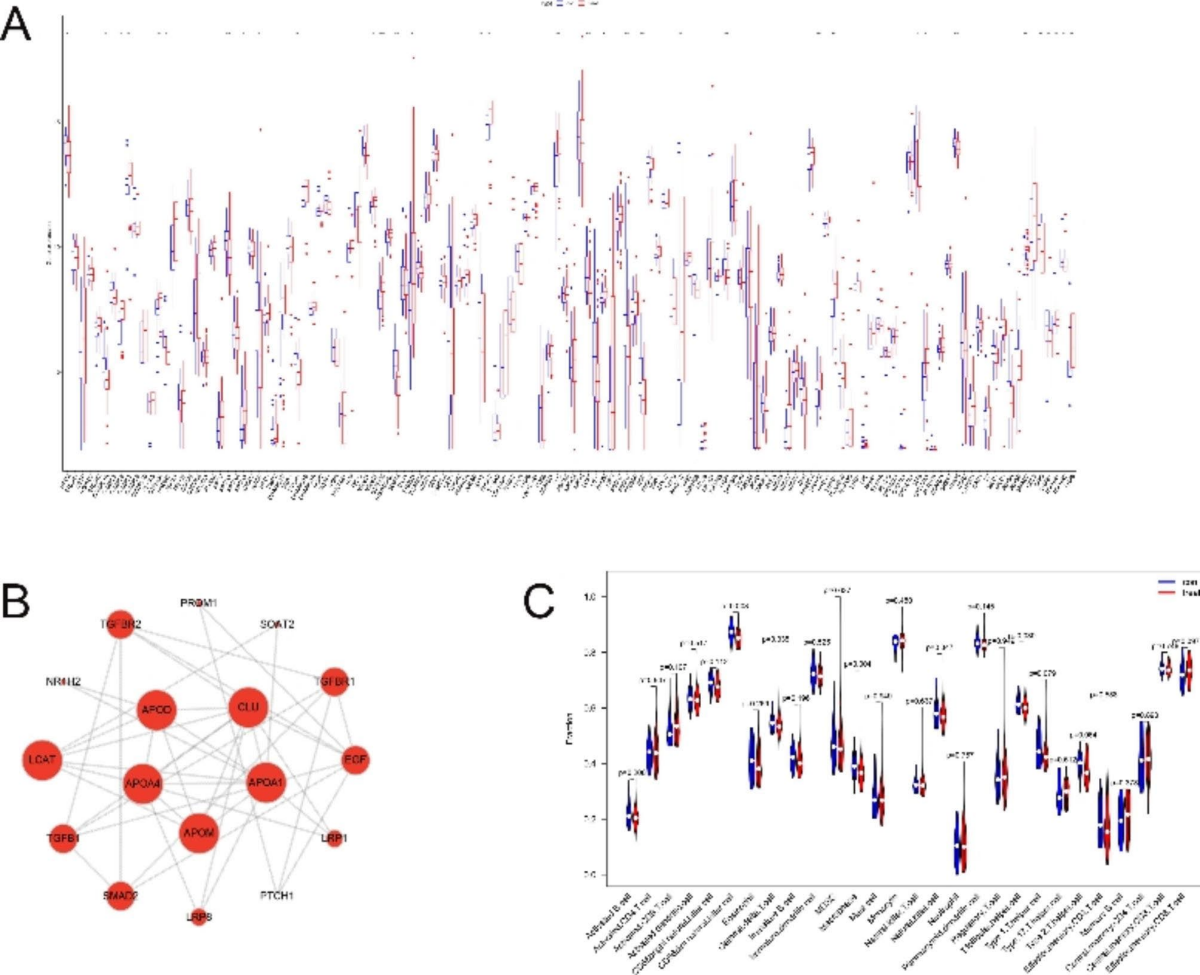
Differential cholesterol metabolism-related related genes. (**A**) 132 cholesterol metabolism-related genes of GSE169498, 34 genes with statistically differential expression in invasive and non-invasive nonfunctioning pituitary neuroendocrine tumors of the cavernous sinus; (**B**) network interaction map of 17 cholesterol metabolism-related genes; (**C**) expression of 28 immune cells in invasive and non-invasive nonfunctioning pituitary neuroendocrine tumors of the cavernous sinus. * represents P < 0.05; * *represents P < 0.01; * * *represents P < 0.001. Treat represents invasive nonfunctioning pituitary neuroendocrine tumors of the cavernous sinus; con represents non-invasive nonfunctioning pituitary neuroendocrine tumors of the cavernous sinus

First, the expression matrix of 132 cholesterol metabolism-related genes in the GSE169498 dataset was acquired. Subsequently, 34 genes that were statistically differentially expressed in invasive and noninvasive NF-PitNETs were further acquired (Fig. 1A). PPI network analysis of 34 genes was performed using the STRING database to obtain the interaction networks of 24 genes, and 17 genes were correlated using the MCODE plug-in of Cytoscape (Fig. 1B).

### Immune cell infiltration assessment by GSE169498

The infiltration of 28 immune cells in the GSE169498 dataset was assessed to explore the relationship between immune cell infiltration and CSI by NF-PitNETs. As shown in Fig. 1C, in invasive and noninvasive NF-PitNETs, only CD56dim natural killer cell, Macrophage, and T follicular helper cell had significant statistical differences, and their *P* values were 0.003, 0.004, and 0.03, respectively. Moreover, the noninvasive group had a higher expression than the invasive group.

### Machine learning

**Fig. 2:**
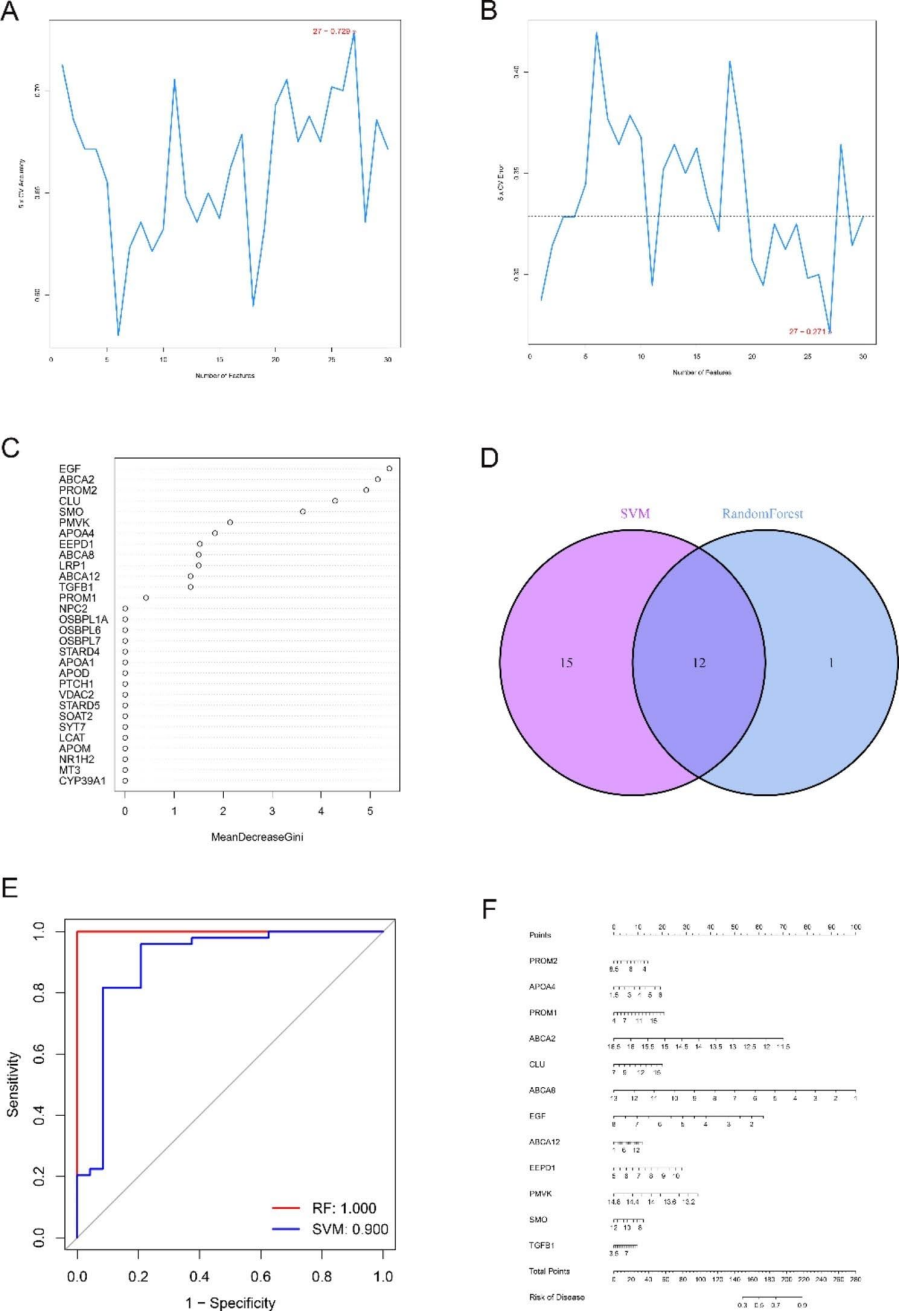
Machine learning for identifying genes involved in cholesterol metabolism. (**A**-**B**) SVM model for screening genes involved in cholesterol metabolism. (**C**) Screening RF models characterizing genes involved in cholesterol metabolism. (**D**) Venn diagram showing the overlap of candidate genes between the above two algorithms. (**E**) ROC curves for SVM model and RF model for 12 hub genes involved in cholesterol metabolism. (F) Nomogram for 12 hub genes. ROC: receiver operating characteristic curve

The RF and SVM algorithms were used to model 34 genes to more accurately screen potential signature genes, and 13 and 27 potential genes were obtained, respectively (Fig. 2A-C). The results were intersected by Venn diagram, and finally, 12 hub cholesterol metabolism-related genes were obtained (Fig. 2D). SVM and RF models were constructed for 12 hub cholesterol metabolism-related genes, with AUC values of 1 for RF models and 0.90 for SVM models (Fig. 2E). Ahub gene-based diagnostic model is constructed to identify nonfunctioning pituitary adenomas invading the cavernous sinus (Fig. 2F).

**Fig. 3:**
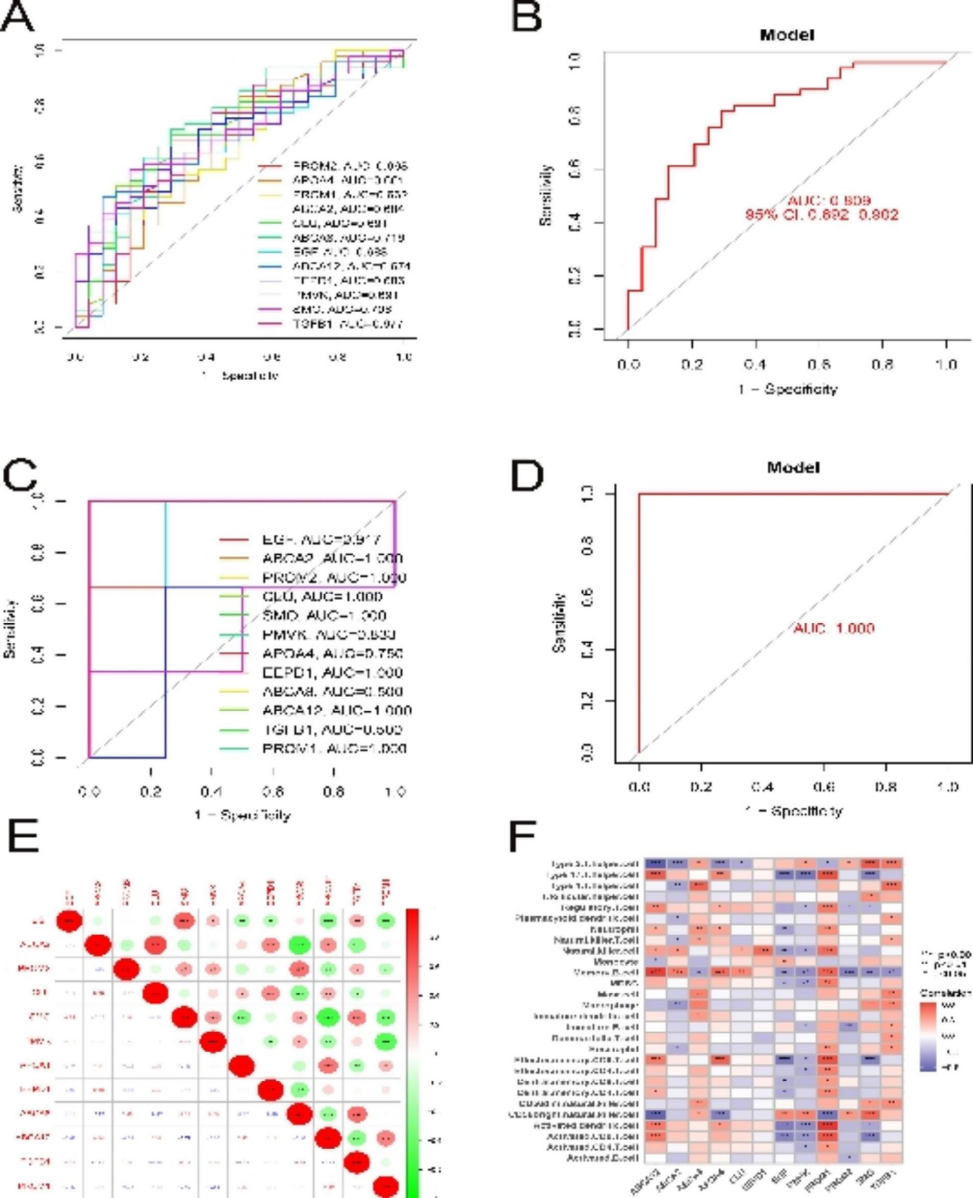
12 hub cholesterol metabolism genes. (**A**) ROC curves for each of the 12 cholesterol metabolism-related genes for the diagnosis of CSI. (**B**) A logistic regression diagnostic model constructed with 12 genes involved in cholesterol metabolism. (**C**) ROC curves for each of the 12 cholesterol metabolism-related genes for the diagnosis of CSI in GSE51618. (**D**) A logistic regression diagnostic model constructed with 12 genes involved in cholesterol metabolism of GSE51618. (**E**) Correlation plots of 12 hub genes involved in cholesterol metabolism; size and color of circles indicate Pearson correlation coefficient. (**F**) GSE169498 data set, relationship plots of cholesterol metabolism-related genes in 28 immune cells at 12 hubs. * represents p < 0.05, * *represents p < 0.01; * * * represents p < 0.001.CSI: cavernous sinus invasion

Subsequently, 12 hub cholesterol metabolism-related genes and logistic regression models were constructed to test the ability of hub genes to identify CSI in the GSE169498 dataset, resulting in AUC values of 0.809 with 95% confidence intervals of 0.692–0.902 (Fig. 3A-B). In the GSE51618 dataset, the diagnostic ability of the model constituted by 12 key genes was beyond compare, and 7 genes had an area under the curve reaching 1 (Fig. 3C-D). Most of the hub genes were statistically significant (*P* < 0.05) from each other (Fig. 3E). As shown in Fig. 3F, memory B cells were significantly associated with 11 genes, and PROM1 was associated with 16 immune cells. In addition, macrophages were associated with ABAA2, ABCA8, SMO, and TGFB1; CD56dim natural killer cell was associated with ABCA8, SMO, and TGFB1; and T follicular helper cell was associated with SMO only.

### Cholesterol-related genes cluster analysis

**Fig. 4:**
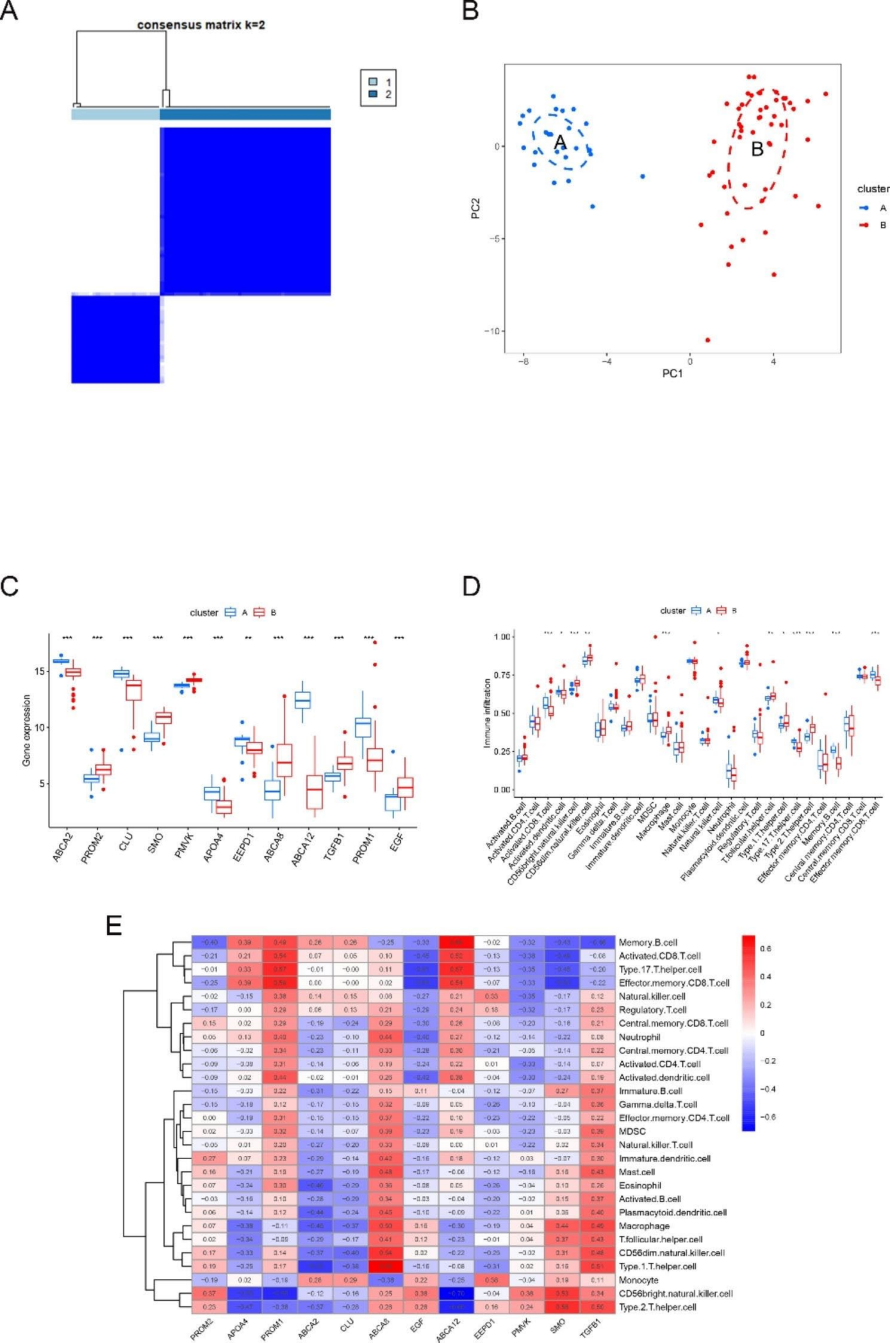
Identification of subtypes of nonfunctioning pituitary neuroendocrine tumors. (**A**) Cluster discrimination using hub genes; (**B**) PCA plots showing the distribution of different sub-clusters; (**C**) expression of 12 hub genes among different subgroups; (**D**) infiltration abundance of 28 immune cells among different subgroups; (**E**) correlation of 28 immune cells among 12 hub genes among different subgroups. PCA, principal component analysis; color indicates Pearson correlation coefficient

The “ConsensusClusterPlus” R package was used to perform consensus cluster analysis of the 12 signature genes, and the results determined that k = 2 provided the most stable grouping (Fig. 4A). Seventy-three samples in the GSE169498 dataset were divided into two distinct classes in consensus cluster analysis, namely, cluster A (n = 25) and cluster B (n = 48). In the two-category PCA analysis, the gene expression patterns differed between clusters (Fig. 4B). As shown in Table 2, a statistical significance could be observed between subgroups A and B and the invasive and noninvasive tumor groups of samples (*P* = 0.001). In both categories, ABCA2, CLU, APOA4, EEPD1, ABCA12, and PROM1 were more highly expressed in group A, and the remaining genes were more highly expressed in group B, as shown in box plots (Fig. 4C). Exploration of the relationship between different clusters and immune cell infiltration demonstrated that 12 immune cells were significantly associated in both groups, with macrophages having *P*-values < 0.0001 and CD56dim natural killer cell and T follicular helper cell having *P*-values < 0.001 in both groups (Fig. 4D). Similarly, most of the 28 immune cells infiltrated more in group A than in group B. However, CD56bright natural killer cell, CD56dim natural killer cell, macrophages, T follicular helper cell, Type 1 T helper cell, Type 2 T helper cell and Effector memory CD4 T cell were more significantly expressed in group B.

### Functional analysis between two subgroups

**Fig. 5:**
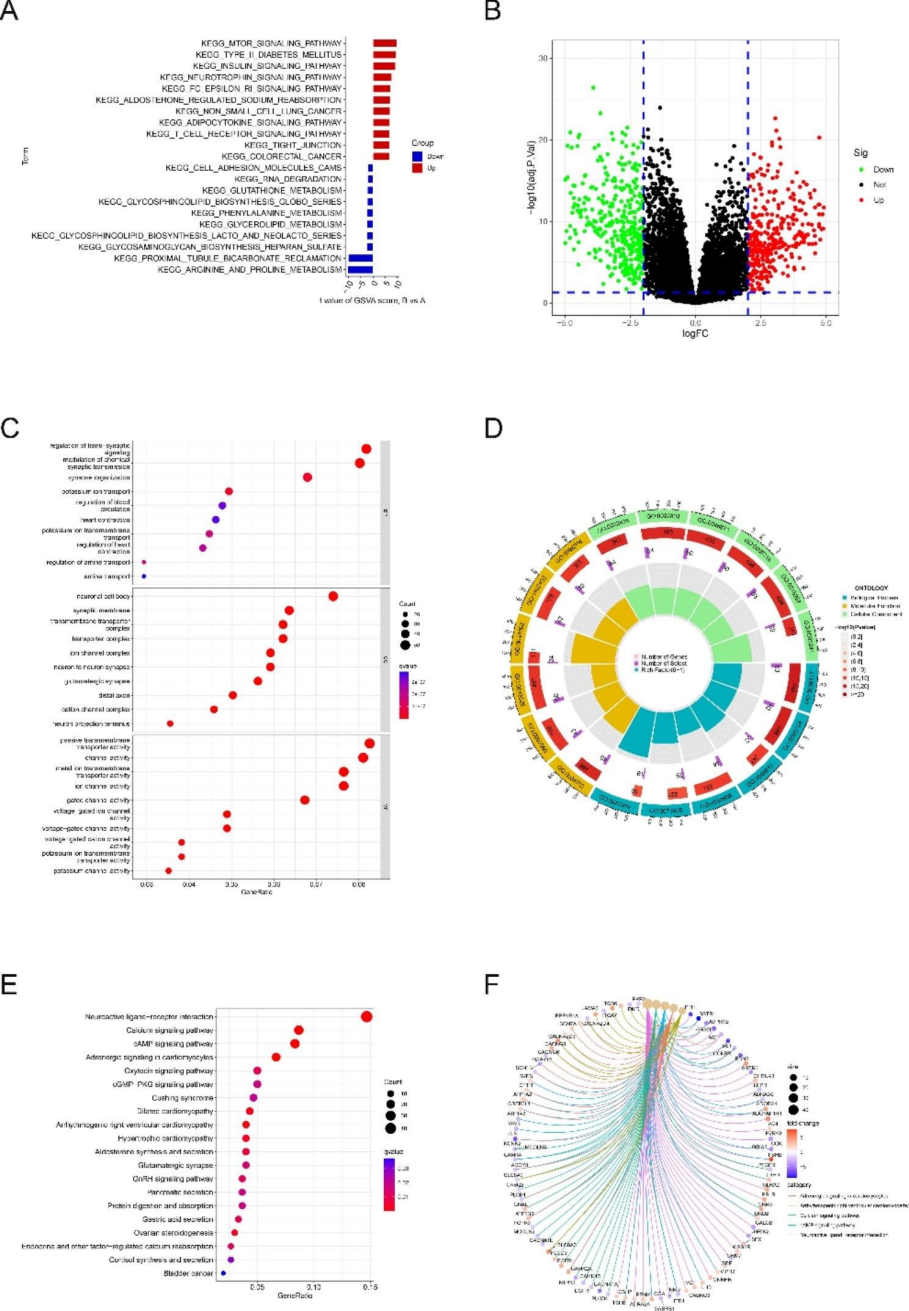
Functional enrichment analysis across subgroups. (**A**) GSVA analysis based on KEGG pathway. (**B**) Volcano plot of differential genes; (**C**-**D**) enriched items in pathway (**E**-**F**) KEGG pathway analysis in GO enrichment analysis

Comparison between the two subgroups by GSVA analysis showed that the mTOR signaling pathway, type II diabetes mellitus, insulin signaling pathway, neurotrophin signaling pathway, and other pathways were upregulated, and the downregulated pathways were mainly arginine and metabolism and proximal tubule bicarbonate reclamation (Fig. 5A).

Subsequently, 743 DEGs were obtained, including 362 upregulated genes and 381 downregulated genes. Their distribution was visualized using volcano plots (Fig. 5B). GO and KEGG enrichment analyses were performed on the 743 DEGs to analyze the molecular function between the two subgroups. In the GO enrichment analysis, BP, CC, and MF fractions were mainly enriched in the regulation of trans-synaptic signaling, neuronal cell body, and transmembrane transporter complex, respectively (Fig. 5C). In the KEGG enrichment analysis, the DEGs were mainly enriched in neuroactive ligand–receptor interaction, calcium signaling pathway, and cAMP signaling pathway (Fig. 5E).

## Discussion

Trans-sphenoidal surgery is the treatment of choice for symptomatic patients with NF-PitNETs, but complete resection of invasive tumors of the cavernous sinus is very difficult [18]. Currently, an increasing number of investigators are focusing on pharmacological treatment options for aggressive NF-PitNETs, such as dopamine agonists, somatostatin receptors, and PI3K/AKT/mTOR pathway inhibitors [18–21]. However, the pharmacological regimen of NFPA remains to be further investigated. Therefore, the present study was the first to analyze the mechanism of cavernous sinus invasive NF-PitNETs from the perspective of cholesterol metabolism to discover potential therapeutic targets that may exist for NF-PitNETs.

Through clinical data studies, we found that the cholesterol concentration in blood and its metabolic process may be associated with CSI in NF-PitNETs. As cholesterol metabolites, bile acids could regulate various aspects of metabolism, apoptosis, proliferation, senescence, and immune environment and affect tumor progression [9]. Ursodeoxycholic acid (UDCA) has been shown to be beneficial against various tumors as an anti-cholestatic or biliary surge [22–24]. Ding et al. demonstrated that dysregulated cholesterol metabolism could promote PitNETs growth by activating hedgehog signaling [13]. Loeper et al. found that Ikaros could have an effect on cell differentiation, maturation, and function by regulating cellular cholesterol homeostasis, which may guide the expansion of pituitary neuroendocrine cells [25]. Similarly, the present study revealed a difference in blood cholesterol concentration between patients with invasive and noninvasive NF-PitNETs. However, the association of this difference with CSI of PA remains to be investigated further.

The immune infiltrates between the invasive and noninvasive groups in the GSE169498 dataset indicated that the abundance of CD56dim natural killer cell, macrophage, and T follicular helper cell infiltration in invasive pituitary neuroendocrine tumors was lower than that in the noninvasive group. Previous studies have suggested that the CD56dim natural killer cells in malignant tumors preferentially undergo apoptosis, which may promote tumor progression [26]. Different subtypes of macrophages have different effects on tumors: the M1 type has antitumor effects, whereas the M2 type has tumor-promoting effects; however, both effects may vary in different tumor stages and types [27]. T follicular helper cells are now emerging as another highly relevant immune cell population in various cancer types, and Gutiérrez-Melo et al. [28] showed that their presence is generally consistent with a better prognosis in solid organ tumor types. This finding is similar to that of the present study, where the infiltration abundance of these three genes was higher in the noninvasive group, and they may have hindered pituitary neuroendocrine tumors from developing aggressive behavior. However, the antitumor and tumor-promoting effects of immune cells may vary at different stages of the tumor, and their effects depend on the specific composition of the tumor immune infiltrate, the overall composition of its TME, and the location of TILs [29].

A total of 34 cholesterol metabolism genes with differential expression in NF-PitNETs were obtained in the invasive and noninvasive groups to investigate the role of cholesterol metabolism-related genes in the CSI by NF-PitNETs. 34 differential cholesterol metabolism-related genes were screened using RF and SVM algorithms, and finally, 12 hub genes (ABCA2, PROM2, CLU, SMO, APOA4, EEPD1, ABCA8, ABCA12, TGFB1, PROM1, EGF, and PMV) were obtained. For further elucidation of the link between cholesterol metabolism-related genes and invasive NF-PitNETs, the associations between 12 cholesterol metabolism genes were identified. Previous studies have shown that all 12 hub genes are associated with tumor progression. ATP-binding cassette (ABC) transporters are a series of transporters that are associated with various biochemical and physiological processes, such as maintaining the cellular environment, preventing harmful substances, and regulating pharmacokinetics [30, 31]. They also play an important role in the progression of various tumors [32]. Inhibition of ABCA2 protein expression has been reported to decrease matrix metalloproteinase expression, thereby inhibiting prostate cancer cell invasion and migration in the TME [33]. ABCA8 and ABCA12 are considered hallmark ABC transporters, which may be involved in the regulation of immune cell infiltration in thyroid cancer [34]. Previous studies have shown that PROM1 is a CSC marker and a regulator of cancer progression and prognosis, and its targeted drugs could hinder the progression of various cancers [35, 36]. Meanwhile, PROM2 and PROM1 are structurally similar [35]. EEPD1 is a structure-specific nuclease that is overexpressed in various tumors, but its mutation in cancer is uncommon, which may help tumor cells manage oncogenic stress or confer resistance to therapeutic agents [37]. APOA4 plays an important role in lipid metabolism and metabolic regulation, and APOA4 protein has been found to be a potential biomarker of malignant tumor differentiation in female tumor serum parameters [38]. Smoothing (Smo), encoded by SMO, is an integral transmembrane protein of Hedgehog (Hh) signaling pathway and plays a critical role in embryogenesis [39]. However, Hh signaling has been demonstrated to promote PitNETs growth. The relevance of these genes in NF-PitNETs has not been documented and requires further investigation. EGF, CLU, and TGFB1 have been reported in studies related to pituitary neuroendocrine tumors. EGF is located in the anterior pituitary gland and is present at all developmental stages from fetus to adulthood [40]. In addition, EGF is expressed in functional and nonfunctional pituitary adenomas, with higher expression in more aggressive tumor subtypes [41]. TGFB1 is an angiogenic growth factor that could show higher levels in pituitary tumors than in normal pituitary tissues [42]. Sheng-Yuan et al. have demonstrated that CLU may be a potential diagnostic biomarker and therapeutic target for invasive NF-PitNETs [43], which further demonstrates that CLU may be one of the factors affecting the development of invasive behavior in PitNETs. However, as an anti-apoptotic and metastasis-promoting factor, the specific mechanism by which CLU promotes CSI by PitNETs remains to be further investigated.

On the basis of the 12 hub genes, two distinct cholesterol metabolism pathway-related sub-clusters were identified using unsupervised cluster analysis. In the subgroup functional analysis, more abundant immune cells were different between the two subgroups than in the invasive and noninvasive groups. This finding suggested that cholesterol metabolism may promote immune infiltration in NF-PitNETs. In addition, DEGs were mainly enriched in neuroactive ligand–receptor interaction and calcium signaling pathway according to the KEGG enrichment analyses. The mTOR signaling pathway was significant based on the GSVA analysis of the contrast between the two subclusters. Neuroactive ligand–receptor interaction and calcium signaling pathway have been found in NF-PitNETs, and they have been revealed to be possibly associated with tumor progression [44, 45], further validating the results of the present study. CCL17 secreted by tumor-associated macrophages could promote pituitary adenoma invasion by enhancing the mTORC1 signaling pathway [46]. Recent studies have also shown that cholesterol is important for activating mTORC1 kinase, and when mTORC1 is activated, it could initiate a program on the lysosomal membrane that upregulates anabolic processes and downregulates autophagy [47].

### Study strengths and limitations

The study’s strength is that it cleverly combined clinical data and GEO database data to find that the association between cholesterol metabolism genes and infiltrating immune cells and the heterogeneity of immune responses in NF-PitNETs patients with sub-clusters of different cholesterol metabolism genes. And, this study established the best diagnostic model to accurately distinguish Invasive and noninvasive NF-PitNETs. This study also has some limitations. First, clinical samples with relatively small sizes were collected, and data from patients with incidentalomas were not included, thus requiring multicenter clinical data to further confirm the role of cholesterol metabolism in the process of CSI by NF-PitNETs. Second, the key genes and important pathways obtained by bioinformatics analysis lack the validation of immunohistochemistry or PCR about hub genes. In addition, GSE169498, the dataset used in this study, defined CSI by Knosp grades on preoperative magnetic resonance images of patients, which may pose some risk to the results.

## Conclusion

The results revealed, for the first time, that enhancing cholesterol metabolism may predispose CSI in NF-PitNETs and provide novel insights into therapeutic strategies for NF-PitNETs. Further, abnormal cholesterol levels can be used as a clinical indicator for early screening, treatment, and monitoring cavernous sinus invasion in cancer patients.

### Electronic supplementary material

Below is the link to the electronic supplementary material.

Supplementary Material 1

Supplementary Material 2

Supplementary Material 3

## Data Availability

The datasets from GSE169498 and the original contributions presented in this study are included in the article material. The names of the repository/repositories and accession number(s) can be found in the article material. Further inquiries can be directed to the corresponding author.

## Abbreviations

AUC: Area Under Curve
CSI: Cavernous sinus invasion
DEGs: The differentially expressed genes
GO: Gene Ontology
GSVA: Gene Set Variation Analysis
KEGG: Kyoto Encyclopedia of Genes and Genomes
NF-PitNETs: Nonfunctioning pituitary neuroendocrine tumors
PCA: Principal components analysis
PitNETs: Pituitary neuroendocrine tumors
PPI: Protein–protein interaction
RF: Random forest
ROC: Receiver operating characteristic curve
ssGSEA: Single sample Gene Set Enrichment Analysis
SVM: Support vector machines
TME: Tumor microenvironment
