# Supplementary material for: Identification of cholesterol metabolism-related subtypes in nonfunctioning pituitary neuroendocrine tumors and analysis of immune infiltration
Source: Lipids Health Dis. 2023 Aug 10;22:127. doi: 10.1186/s12944-023-01883-3 (PMC10413501; doi:10.1186/s12944-023-01883-3)
Supplement: Supplementary file 1 — Supplementary Material 1 [file 12944_2023_1883_MOESM1_ESM.pdf]

ShineWrite.com
SINCE 2006

Certificate of English Editing

To whom it may concern:

This memo certifies that one of our clients had contracted our academic editing service for the following file (s).

Order Number:
40-2023030120193336

Word Count:
3825 ~ 4250 words

Date of the review:
3/1/2023 (MM/DD/YYYY)

The English review was conducted using a two-stage process in which a junior editor first reviewed the file, and then a senior editor conducted the final and more thorough review. All our editors are native English speakers.

We would like to emphasize that our service is targeted on grammar and language edits. We do not rewrite the documents from scratch. If you feel certain points are incorrect, please call our attention by contacting service@shinewrite.com

Documents receiving this certification should be English-ready for publication - however, the author has the ability to accept or reject our suggestions and changes.

Ken Galloway

Editor in charge
